# Selective IgM Hypogammaglobulinemia and Multiple Sclerosis Treated with Natalizumab and Ofatumumab: A Case Report

**DOI:** 10.3390/jpm15040155

**Published:** 2025-04-17

**Authors:** Francesco Crescenzo, Michelangelo Turazzini, Francesca Rossi

**Affiliations:** Neurology Unit—Multiple Sclerosis Center, “Mater Salutis” Hospital, Verona Local Health Authority of Veneto Region (AULSS 9 Scaligera), 37045 Legnago, Italyfrancesca.rossi@aulss9.veneto.it (F.R.)

**Keywords:** multiple sclerosis, hypogammaglobulinemia, disease-modifying treatment, anti-CD20

## Abstract

**Background:** B-cell-depleting drugs targeting the CD20 antigen have been increasingly implemented as an “exit strategy” from natalizumab for relapsing–remitting multiple sclerosis (RRMS) patients due to the increased risk of progressive multifocal leukoencephalopathy. Data on recently approved anti-CD20 drugs, such as ofatumumab serving as a natalizumab “exit strategy”, are lacking. Furthermore, due to their immunosuppressive mechanism of action, prolonged use of these “highly effective” drugs is associated with the development of hypogammaglobulinemia and, consequently, a higher risk of infections. There are no guidelines for monitoring serum immunoglobulin levels in individuals undergoing “highly effective” multiple sclerosis treatment. **Methods:** We present a case of a 26-year-old male RRMS patient with selective IgM deficiency and multiple sclerosis initially treated with natalizumab and later ofatumumab. **Results:** The patient achieved “no evident disease activity “status while undergoing treatment with natalizumab and ofatumumab, but these therapies, especially ofatumumab, greatly impacted further drops in IgM levels. However, no significant decrease in IgG levels was observed, and no infectious events occurred. In addition, the patient did not show signs of disease activity while on ofatumumab, which also offered a more convenient mode of administration. **Conclusions**: Our experience points to the need to further explore benefit–risk ratios of highly effective treatments, even in cases with low immunoglobulin levels. However, closely monitoring immunoglobulin levels and conducting clinical follow-ups to ensure prompt recognition of potential infectious complications constitute approaches that have been thought of for such patients.

## 1. Background

Multiple sclerosis (MS) is a chronic, inflammatory, demyelinating, neurodegenerative disease that affects the central nervous system (CNS) and is more common in young adults [1].

Many specific disease-modifying therapies (DMTs) are currently available for MS. They are commonly distinguished as moderately or highly effective DMTs [2]. Several studies have shown that early use of highly effective DMTs reduces the risk of accumulation of irreversible clinical disability [3,4]. Therefore, they should be promptly proposed to young patients, especially in the context of long-term unfavorable prognostic factors (e.g., male sex; functional multisystem involvement; short inter-relapse latency in the first 2 years; high lesion load; the presence of gadolinium-enhancing lesions; the presence of brainstem, cerebellar, and spinal cord lesions; persistent disease activity while undergoing DMT; and the presence of cerebrospinal oligoclonal bands).

The decision to employ which of the different DMTS should also be based on each patient’s drug administration preferences, needs, and lifestyles [5].

Over the last few years, monoclonal antibody therapies targeting the CD20 antigen on B-cells (rituximab, ocrelizumab, ofatumumab, and ublituximab) have been increasingly employed in treating MS, providing high efficacy and a favorable benefit–risk profile [6]. Ofatumumab is the first approved subcutaneously and monthly self-injected anti-CD20 drug to expand the therapeutic scenario for MS to intensive treatment of this disease and ensure each patient’s independence and convenience [7].

Beyond being a pharmacological option for MS patients with negative predictive factors, anti-CD20 DMTs are currently widely used as treatment-switch strategies with respect to other highly DMTs, particularly for patients shifting from natalizumab because of an increased progressive multifocal leukoencephalopathy (PML) risk [8]. However, considering their distinct pharmacological properties, the best anti-CD20 option, as with the best natalizumab “exit strategy”, is uncertain, and data on recently approved drugs (ofatumumab and ublituximab) are lacking.

Furthermore, due to their B-cell-depleting mechanism of action, the prolonged use of anti-CD20 monoclonal antibodies could induce hypogammaglobulinemia and pose a higher risk of infection [9]. Nevertheless, the prevalence of hypogammaglobulinemia appears to differ among the various anti-CD20 drugs [10]

To better understand the benefit–risk ratio of highly effective treatment with ofatumumab as a natalizumab-exit strategy, we present a case of an MS patient with partial selective IgM deficiency treated with natalizumab for one year and later with ofatumumab.

## 2. Case Report

In December 2020, a 26-year-old man with a family history of maternal MS presented to our clinic complaining of diplopia. The first neurological evaluation revealed external ophthalmoplegia due to right oculomotor nerve paresis. No other sensorimotor abnormalities in other cranial nerves, limbs, or trunks were observed; the brain magnetic resonance imaging (MRI) results were negative, and the results of blood analysis, screening tests for rheumatologic disease, and thyroid function test were within normal limits, although low serum IgM levels were observed (40 mg/dL l.n. 50–300 mg/dL). Because of a diagnostic suspicion of idiopathic (inflammatory) cranial mononeuropathy, the patient received high-dose oral steroid therapy (prednisone 1 mg/kg/day) for ten days, followed by gradual tapering, with complete resolution in three weeks. The follow-up visit 4 weeks after the clinical onset showed a complete resolution of the disorder, and a second confirmatory blood analysis panel confirmed mild hypogammaglobulinemia M.

In March 2021, the patient returned to our clinic because he was experiencing multifocal neurological symptoms. He suffered from sensations of distal leg pins and needles, visual blurring in the right eye, and the recurrence of diplopia. No fever or flu-like symptoms were reported in the previous days. We obtained a fresh contrast-enhanced MRI of the brain and spinal cord, and the results were consistent with demyelinating lesions typical of MS at hemispheric, optic nerve, infratentorial, and spinal cord levels. The results of cerebrospinal fluid analysis were positive for oligoclonal bands. Therefore, the patient was diagnosed with MS [11] (Expanded Disability Status Scale—EDSS 2). The patient made a complete recovery following acute treatment with high-dose intravenous steroids (EDSS 0). Although the patient’s anti-John Cunningham virus (JCV) antibody status was positive, with an index >1.5, a highly effective DMT with natalizumab was initiated early. After six months of clinical stability, the treatment was transitioned from a 4-week standard interval dosing regime to a 6-week extended dosing regime to mitigate the risk of PML [12].

While undergoing natalizumab treatment, the patient showed no evidence of the three clinical/imaging measures of disease activity (NEDA-3 i.e., no clinical relapse, new MRI lesions, or disability progression) [13], and a further slight reduction in serum IgM levels was observed (34 mg/dL l.n. 50–300 mg/dL).

His quality of life and graduate studies were pleasing and satisfactory, so he went abroad to complete his postgraduate program 12 months after starting natalizumab. Since frequent natalizumab intravenous administration interfered with his study planning and he had a high JCV antibodies index, we decided to switch the treatment to ofatumumab. In April 2022, after a 6-week interval between the last administration of natalizumab, the patient started a course of ofatumumab. Re-baseline MRI at 3 months did not show signs of disease reactivation. No further monitoring of the anti-JCV index was carried out.

**Figure 1:**
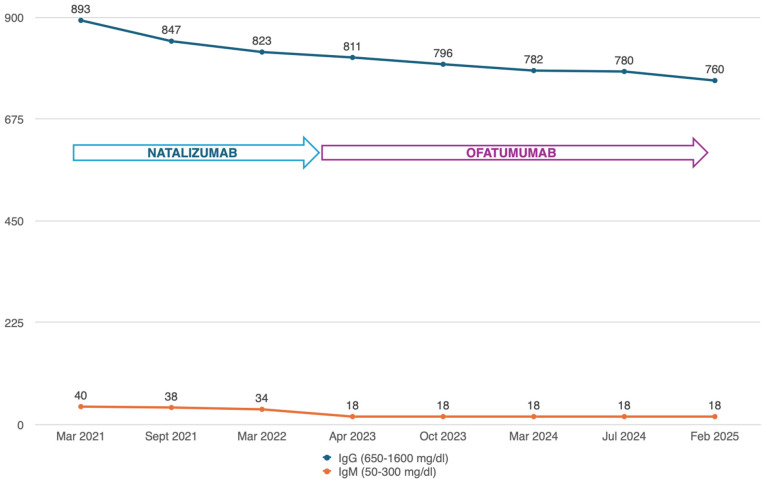
The graph shows the trend regarding immunoglobulins M and G over the years during therapy with natalizumab and then ofatumumab.

The patient sustained NEDA-3-status treatment in these three years while undergoing ofatumumab, which was highly accepted and well-tolerated. The only cause of concern was marked IgM hypogammaglobulinemia: during the first year of ofatumumab, the IgM value dropped further (18 mg/dL), satisfying the criteria for clinically relevant hypogammaglobulinemia, formally defined as significant (<2-standard-deviation-decreased serum concentrations of at least one of the three main immunoglobulin isotypes, i.e., IgG, IgA, and IgM) [14]. A slight decrease in IgG levels was also recorded, but they were still within the normal range (Figure 1).

Because of a suspicion of an inherited haematological disorder, namely, common variable immunodeficiency (CVID), we also retrospectively investigated the humoral immunological profile of the patient’s mother, who was 52 years old and also had a history of mild IgM hypogammaglobulinemia (49 mg/dL l.n. 50–300 mg/dL).

Both mother and son reported having an adequate immune response to hepatitis B, measles–mumps–rubella, and varicella-zoster vaccines administered before starting the DMTs or being diagnosed with MS. So far, neither of them has experienced infectious adverse events.

A subsequent hematological consultation ruled out the hypothesis of affliction with CVID, a heterogeneous genetic disorder that affects the immune system and leads to hypogammaglobulinemia and increased susceptibility to recurrent infections, the diagnosis of which requires at least a marked drop in IgG levels and a poor response to vaccines.

However, we decided to continue treating the patient with ofatumumab with routine clinical and laboratory monitoring. No infectious events have occurred over 3 years of follow-up.

## 3. Discussion

This simple clinical case highlights some critical aspects of managing MS patients. Our report reflects a case of selective IgM deficiency, a primary immunological disorder with a prevalence of 0.37% in the general population [15], but it is also reported to be associated with the development of autoimmune diseases [16]. Also, the prevalence of CVID (0.3%) among MS patients is higher than in the general population (0.01%) [17]. However, our case does not meet the diagnostic criteria for CVID [18].

In our case, the evidence of low IgM levels was found at baseline “serendipitously”. In this regard, the value of humoral immunological status as a part of diagnostic and therapeutic workups for MS patients is largely debated. A previous study suggests that MS may contribute to hypogammaglobulinemia [17,19], a hypothesis not confirmed in another study, which also reported a lack of consistency regarding the relationship between this disease, DMTs, immunoglobulin levels at baseline, and the risk of future infection [20].

Concerning DMTs, one recent study also showed that except for platform therapies, moderately and highly effective DMTs are generally associated with reduced immunoglobulin levels, with a declining trend over the course of treatment [17]. Specifically for natalizumab, Selter et al. demonstrated a reduction of up to 30% of serum IgM levels during the first year compared with the level before treatment initiation [21], in line with what we observed for our patients.

Ofatumumab treatment mostly impacted further IgM reduction; IgG levels also decreased but remained within normal limits. This drug, administered subcutaneously, does not induce a significant depletion of IgG-expressing B cells in the spleen, unlike other anti-CD20 therapies administered intravenously [22]

In ASCLEPIOS I/II phase-III randomized controlled trials [23], which compared the efficacy and safety of ofatumumab, with teriflunomide as an active comparator, patients with IgM/IgG hypogammaglobulinemia at baseline were excluded. In addition, the required drop-out (according to protocols) of 65 (3.3%) of the total enrolled 1969 patients in the ASCLEPIOS I/II, APLIOS, APOLITOS, and ALITHIOS trials whose IgM levels have dropped ≥10% below the lower limits of normal could have limited the ability to detect hypogammaglobulinemia safety-related issues [24].

Overall, previous data on the safety of anti-CD20 have demonstrated that IgG hypogammaglobulinemia is linked with a significant risk of infection rather than low IgM levels [25]. In addition, data analysis from the ALITHIOS 4-year extension study revealed no associations between decreased Ig levels and the risk of infection [26] in the presence, as demonstrated in the ASCLEPIOS trials, of a high efficacy of ofatumumab leading to both >50% and >30% reductions in annualized relapse rates and disability progression at 6 months, respectively [23]. Furthermore, a network meta-analysis showed that ofatumumab is not different from the most highly effective DMTs in this regard, such as alemtuzumab, ocrelizumab, and natalizumab [27].

Although subgroup analysis of 57 patients transitioning from natalizumab to ofatumumab in the ASCLEPIOS trials is lacking, growing evidence from real-world studies suggests that B-cell-depleting DMTs are the preferred “exit strategy” for patients with an increased risk of developing natalizumab-associated PML due to these therapies’ optimal efficacy–safety balance in terms of preventing rebound disease activity and PML occurrence [28]. The optimal transition interval has been determined to be 4–8 weeks [29], as implemented for our patient.

A recent prospective comparative multicenter study comparing ofatumumab and ocrelizumab produced intriguing results, showing potential superiority in terms of disease control for patients who switched from natalizumab to ocrelizumab. Nevertheless, the subgroup analysis based on the “washout period” for the same cohort showed no significant differences between the two treatments [30]. These preliminary data can help clinicians to continue to tailor anti-CD20 treatment further, even if it requires stopping natalizumab therapy, to a given patient’s needs, particularly for those with dynamic lifestyles, for whom ofatumumab was reported to ensure independence from infusion and, for an outpatient, lead to a positive perception of their disease and a subjective improvement in their quality of life, as also demonstrated in a recent observational real-world study [31].

## 4. Conclusions

We believe sharing real-world cases like this can help us better understand the benefit–risk ratio of highly effective DMTs, even for abnormalities in serum immunoglobulin values and no clinical correlations. Likewise, our experience reveals the beneficial impact of employing a “rapid switch” from natalizumab to avoid disease reactivation and points out the role of IgG as the most important immunoglobulin for protective immunity, especially while undergoing treatment with B-cell-targeting drugs, whose use can also lead to a reduction in IgM levels without impacting safety.

No consensus guidelines for monitoring serum immunoglobulin levels during disease-modifying treatments are currently available. For patients with asymptomatic IgM and/or IgG hypogammaglobulinemia, active surveillance, along with laboratory monitoring (e.g., every 6 months) and implementing a multidisciplinary clinical evaluation, is recommended [32].

However, further prospective real-world studies are needed to help identify the most accurate patient profile for ofatumumab and evaluate the treatment’s longer-term impact on safety performance.

## Data Availability

The original contributions presented in this study are included in the article. Further inquiries can be directed to the corresponding author.
